# Clinical and epidemiological characteristics and associated factors of hair graying: a population-based, cross-sectional study in Turkey^☆^^☆☆^

**DOI:** 10.1016/j.abd.2020.03.002

**Published:** 2020-03-20

**Authors:** Ersoy Acer, Didem Arslantaş, Gülsüm Öztürk Emiral, Alaattin Ünsal, Burcu Işıktekin Atalay, Saniye Göktaş

**Affiliations:** a Department of Dermatology, Faculty of Medicine, Eskişehir Osmangazi University, Eskişehir, Turkey; b Department of Public Health, Faculty of Medicine, Eskişehir Osmangazi University, Eskisehir, Turkey

**Keywords:** Cross-sectional studies, Epidemiology, Hair color, Prevalence

## Abstract

**Background:**

Hair graying is common in humans; but there is scarce data about its epidemiology.

**Objective:**

This study aimed to evaluate the clinical and epidemiological characteristics and associated factors of hair graying.

**Methods:**

A total of 1541 volunteers between 15 and 65 years old were included in this population-based, cross-sectional study. A questionnaire on characteristics and associated factors of hair graying was filled in by face-to-face interview method.

**Results:**

One thousand sixty three participants (69.0%) had hair graying. The mean onset age of hair graying was 32.9 ± 9.8 years. It was 31.7 ± 9.5 years in females, whereas 33.7 ± 10.0 years in males (*p* = 0.001). The most common involved area of hair graying at the onset and at the time of the interview was temporal region. When it was evaluated by gender, it was temporal in males whereas parietal in females. Hair graying was more severe in males than in females and in late-onset hair graying than early-onset hair graying (respectively, *p* = 0.000, *p* < 0.001). The most common involved area at the onset and at the present was temporal in severe hair graying; whereas parietal in mild hair graying. In logistic regression analysis, age, educational status, presence of hair loss, skin type, family history of early-onset hair graying and anxiety were independently related to hair graying (*p* < 0.05).

**Study limitations:**

The study was performed in only Turkish individuals. The recall biases were another limitations.

**Conclusion:**

Male gender, late-onset and temporal-onset of hair graying may be considered to be poor prognostic factors for hair graying. There is need for further epidemiological studies in people with different ethnic origin to illuminate the clinical and epidemiological characteristics and associated factors of hair graying.

## Introduction

Hair Graying (HG) is a process of chronological aging, and occurs in varying severity regardless of gender and race.^1^, ^2^ Genetic and environmental factors, nutritional status and oxidative stress play roles in the etiopathogenesis of HG.^2^, ^3^, ^4^ In previous studies, it was reported that early-onset HG is associated with a variety of factors such as family history, smoking, alcohol consumption, diet, atopy, obesity, dyslipidemia, and various systemic diseases such as coronary artery diseases, osteopenia and hypothyroidism.^5^, ^6^, ^7^, ^8^, ^9^, ^10^, ^11^, ^12^, ^13^

There is scarce data related with prevalence, clinical and epidemiological characteristics and associated factors of HG although it affects the majority of people throughout life. This study aimed to evaluate the prevalence, clinical and epidemiological characteristics and associated factors of HG in Turkish population.

## Methods

This is a population-based, cross sectional study, conducted in Beylikova and Alpu district centers of Eskişehir city in Turkey between December 2017 and January 2018. Each rural districts of Ekişehir (total 12 districts) was accepted as a cluster for the planned study in the rural settlement of Eskişehir. Alpu and Beylikova districts were determined by lottery method as the working area. According to Turkish Statistical Institute, data population with 15–65 years of Beylikova district was 6589 [3337 (50.6%) male, 3252 (49.4%) female] and 2300 (35%) lived in the district center. Population with 15 to 65 years of Alpu district was 7411 [3880 (52.4%) male, 3531 (47.6%) female] and 2200 (29.7%) lived in the district center.^14^ The frequency of HG was accepted as 50% and minimum sample size was calculated as 1484 with 5% type 1 error (alpha), 95% confidence interval and 3.5% acceptable margin of error. One thousand five hundred and forty-one individuals (15–65 years) were included in the study. The ethnic origins of all participants were Turkish.

Ethics committee approval was obtained before the study (decision no. 2017/04). During the study, the residences in both district centers were visited door to door. Informed consent was obtained from all participants.

A questionnaire evaluating clinical and epidemiological characteristics of HG and socio-clinical risk factors associated with HG and Depression Anxiety Stress scale 21 (DASS 21) were filled in by face-to-face interview method. Individuals who reported to have HG were examined and confirmed by a physician. Involved areas of HG at the present were recorded. If there is ≥1 white hairs, it is considered as HG. The number of graying hairs was classified as <10, 10–100, and >100. Participants with hypopigmentation disorders, alopecia other than androgenetic alopecia, who dyed their hair in the last 3 months and individuals who refused to participate in the study, were excluded. Participants with HG were included in the group with HG and the others were included in the group without HG. Age of onset of HG and involved area of HG at the onset were asked in the questionnaire. Participants with onset age of HG before the age 20 years were included in the early-onset HG group and the others were included in the late-onset HG group.

Status of depression, anxiety and stress were evaluated with DASS-21 which developed from DASS-42.^15^, ^16^ The validity and reliability of the Turkish version of the DASS-21 was conducted by Yılmaz et al.^17^ DASS-21 consisted of 21 questions scored between 0 and 3. The scores given for each item in the scale are collected; then the score is multiplied by 2. Higher total scores on this scale suggest higher depression, anxiety and stress levels. Depression, anxiety and stress levels are divided into 5 categories: normal, mild, moderate, severe and very severe.^15^, ^16^

### Statistical analysis

IBM SPSS Statistics 21.0 program was used for the data analyses. Continuous data was presented as mean ± standard deviation. Categorical data was presented in percentage (%) values. Mann Witney *U*, Pearson Chi-Square analysis and Backward Stepwise Logistic Regression Analysis was used. *p* ≤ 0.05 values was accepted to be statistically significant.

## Results

Of 1541 (32.4% of total population of Alpu and Belikova district) participants age range 15–65 years, mean 40.4 ± 14.5 years, 886 (57.5%) male, 655 (42.5%) female were included in the study. Of all participants, 765 (33.3% of total population of Beylikova district) were from Beylikova district and 776 (35.3% of total population of Alpu district) were from Alpu district. Of 1541 participants, 1063 (69.0%) had HG, 478 (31.0%) did not. Of 1063 participants with HG, 437 (41.1%) were female, 626 (58.9%) were male. The prevalence of HG increased with age (Table 1) (*p* = 0.00). The mean onset age of HG was 32.9 ± 9.8 years (range 10–60 years). It was 31.7 ± 9.5 years in females, whereas 33.7 ± 10.0 years in males (*p* = 0.001). Of 1063 participants with HG, 80 (7.5%) had early-onset HG and 983 (92.5%) had late-onset HG. Lower monthly income and educational status, higher body mass index, darker skin type, presence of chronic disease, hair loss, family history of early-onset HG and anxiety were significantly higher in subjects with HG (*p* < 0.05) (Table 1).

**Table 1:** Sociodemographic characteristics of participants with HG and without HG

Hair graying			*p*-value^a^
Characteristic	Absent *n* (%)	Present *n* (%)	
*Gender*			0.099
Female	218 (33.3)	437 (66.7)	
Male	260 (29.3)	626 (70.7)	

*Age*			0.000
≤20	100 (86.2)	16 (13.8)	
21–30	221 (59.7)	149 (40.3)	
31–40	101 (32.3)	212 (67.7)	
41–50	44 (14.8)	254 (85.2)	
≥51	12 (2.7)	432 (97.3)	

*Educational status*			0.000
Primary school	99 (15.7)	531 (84.3)	
High school	203 (43.3)	266 (56.7)	
University	176 (39.8)	266 (60.2)	

*Marital status*			0.000
Single	267 (61.2)	169 (38.8)	
Married	200 (19.3)	834 (80.7)	
Widow/widower	11 (15.5)	60 (84.5)	

*Monthly income, Turkish Lira*			0.001
<2000	30 (19.7)	122 (80.3)	
2000–6000	323 (30.8)	725 (69.2)	
>6000	125 (36.7)	216 (63.3)	

*Smoking*			0.447
Yes	180 (29.9)	422 (70.1)	
No	298 (31.7)	641 (68.3)	

*Alcohol consumption*			0.000
Present	121 (41.0)	174 (59.0)	
Absent	357 (28.7)	889 (71.3)	

*Diet*			0.803
Vegetarian	44 (28.8)	109 (71.2)	
Mostly meat	86 (30.8)	193 (69.2)	
Mixed	348 (31.4)	761 (68.6)	

*BMI*			0.000
<18.5	320 (42.9)	426 (57.1)	
18.5–24.9	131 (22.2)	458 (77.8)	
≥25	27 (13.1)	179 (86.9)	

*Fitzpatrick's skin type*			0.005
1/2	136 (34.9)	254 (65.1)	
3	166 (27.7)	433 (72.3)	
4	119 (36.1)	211 (63.9)	
5	57 (25.7)	165 (74.3)	

*Chronic disease*			0.000
Absent	389 (40.1)	581 (59.9)	
Present	89 (15.6)	482 (84.4)	

*Hair loss*			0.000
Absent	257 (37.4)	431 (62.6)	
Present	221 (25.9)	632 (74.1)	

*Dandruff*			0.175
Absent	362 (30.2)	838 (69.8)	
Present	116 (34.0)	225 (66.0)	

*Hair dye history*			0.065
Absent	395 (32.1)	835 (67.9)	
Present	83 (26.7)	228 (73.3)	

*Family history of early-onset HG*			0.000
Absent	307 (46.7)	350 (53.3)	
Present	171 (19.3)	713 (80.7)	

*Depression*			0.208
Absent	333 (32.1)	706 (67.9)	
Present	145 (28.9)	357 (71.1)	

*Anxiety*			0.012
Absent	321 (33.3)	643 (66.7)	
Present	157 (27.2)	420 (72.8)	

*Stress*			0.296
Absent	368 (31.7)	792 (68.3)	
Present	110 (28.9)	271 (71.1)	

*Total*	478 (31.0)	1063 (69.0)	

HG, hair graying; BMI, body mass index.

a Pearson chi-square.

Of 1063 participants with HG, 606 (57.0%) had more than 100 gray hairs, 280 (26.3%) had 10–100 gray hairs, 177 (16.7%) had fewer than 10 gray hairs. Severity of HG increased with age (*p* = 0.000). HG was more severe in males than in females (*p* = 0.000) (Table 2). In addition, it was more severe in participants with late-onset HG than in participants with early-onset HG (*p* < 0.001) (Table 3).

**Table 2:** Characteristics of participants with HG according to gender

Characteristic	Gender		*p*-value
	Female *n* (%)	Male *n* (%)	
*Severity of HG*			0.000^a^
<10	95 (21.7)	82 (13.1)	
10–99	125 (28.6)	155 (24.8)	
>100	217 (49.7)	389 (62.1)	
Total participants with HG	437 (100)	626 (100)	

*Involved area at the onset*^b^			
Frontal	143 (32.7)	110 (17.5)	
Parietal	228 (52.1)	170 (21.1)	
Temporal	133 (30.4)	468 (74.7)	
Occipital	54(12.3)	87 (13.8)	

*Involved area at the present*^b^			
Frontal	261 (59.7)	381 (60.8)	
Parietal	345 (78.9)	402 (64.2)	
Temporal	281 (64.3)	524 (83.7)	
Occipital	211 (48.2)	366 (58.4)	

a Pearson chi-square.

b Participants may have more than one area. HG, hair graying.

**Table 3:** Characteristics of participants with HG according to age of onset

Characteristic	Age of onset of HG		*p*-value
	≤19 *n* (%)	≥20 *n* (%)	
*Severity of HG*			<0.001^a^
<10	29 (36.3)	148 (15.1)	
10–100	23 (28.8)	257 (26.1)	
>100	28 (35.0)	578 (58.8)	
Total participants with HG	80 (100)	983 (100)	

*Involved area at the onset*^b^			
Frontal	16 (20)	237 (24.1)	
Parietal	46 (57.5)	352 (35.8)	
Temporal	25 (31.2)	581 (59.1)	
Occipital	11 (13.7)	126 (12.8)	

*Involved area at the present*^b^			
Frontal	38 (47.5)	621 (63.1)	
Parietal	61 (76.2)	772 (78.5)	
Temporal	48 (60)	901 (91.6)	
Occipital	34 (42.5)	546 (55.5)	

a Pearson chi-square.

b Participants may have more than one area. HG, hair graying.

The most common involved area of HG at the onset and the present was temporal region in all participants with HG. When it was evaluated by gender, it was temporal region in males whereas parietal region in females (Table 2). Occipital region was the least involved area in both genders. According to the onset age of HG, the most common involved area of HG at the onset and the present was parietal region in early-onset HG whereas temporal region in late-onset HG (Table 3). According to the severity of HG, the most common involved area of HG at the onset and the present was parietal region in participants with mild HG whereas temporal region in participants with severe HG (Table 4).

**Table 4:** Characteristics of participants with HG according to severity of HG

Characteristic	Severity of HG		
	<10 *n* (%)	10–100 *n* (%)	>100 *n* (%)
*Total participants with HG*	177 (100)	280 (100)	606 (100)

*Involved area at the onset*^a^			
Frontal	28 (15.8)	60 (21.4)	165 (27.2)
Parietal	94 (53.1)	95 (33.9)	209 (34.4)
Temporal	66 (37.2)	154 (55)	386 (63.6)
Occipital	17 (9.6)	26 (9.2)	94 (15.5)

*Involved area at the present*^a^			
Frontal	39 (22)	101 (36)	519 (85.6)
Parietal	99 (55.9)	139 (49.6)	529 (87.2)
Temporal	71 (40.1)	191 (68.2)	569 (93.8)
Occipital	21 (11.8)	65 (23.2)	492 (81.1)

a Participants may have more than one area. HG, hair graying.

In univariable and multivariable logistic regression analysis, age, educational status, hair loss, darker skin types, family history of early-onset HG and anxiety were independently related to HG (*p* < 0.05) (Table 5).

**Table 5:** Odds of HG according to univariable/multivariable logistic regression analysis

Variables	Univariable analysis		Multivariable analysis (final step 6)	
	*β*	OR (CI)	*β*	OR (CI)
*Age (ref. ≤20)*				
21–30	1.36	3.91 (2.72–5.62)^c^	1.44	4.21 (2.39–7.43)^c^
31–40	2.38	10.77 (7.23–16.05)^c^	2.57	13.12 (7.36–23.40)^c^
41–50	3.55	34.78 (21.33–56.72)^c^	3.59	36.08 (19.46–66.88)^c^
≥51	5.03	153.30 (71.6–328.13)^c^	5.42	225.00 (103.20–490.55)^c^

*Educational status (ref. university)*				
High school	1.27	3.55 (2.66–4.73)^b^	−0.468	0.63 (0.44–0.89)^b^
Primary school	0.143	0.87 (0.67–1.13)	−0.266	0.77 (0.16–1.12)

*Hair loss (ref. absent)*				
Present	0.53	1.71 (1.37–2.12)^c^	0.35	1.42 (1.06–1.90)^a^

*Fitzpatrick's skin type (ref. type 1/2)*				
Type 3	0.33	1.40 (1.06–1.84)^a^	0.61	1.84 (1.26–2.70)^b^
Type 4	−0.05	0.95 (0.70–1.29)	0.06	1.10 (0.70–1.62)
Type 5	0.44	1.55 (1.08–2.24)^a^	0.82	2.26 (1.38–3.69)^b^

*Family history of early-onset HG (ref. absent)*				
Present	1.30	3.66 (2.92–4.59)^c^	1.69	5.40 (3.98–7.34)^c^

*Anxiety (ref. absent)*				
Present	0.29	1.34 (1.06–1.68)^a^	0.29	1.34 (0.98–1.83)

HG, hair graying.

a *p* < 0.05.

b *p* < 0.01.

c *p* < 0.001.

Of 1063 participants with HG, 482 (45.3%) had at least one history of chronic disease. These were hypertension (28.3%), thyroid diseases (14.2%), cardiovascular diseases (13.8%), respiratory system diseases (12.2%), lipid metabolism diseases (10.3%), anemia (9.8%), diabetes mellitus (9.7%) and cancer (1.7%), respectively.

## Discussion

In the literature there are scarce data about prevalence, clinical and epidemiological characteristics and associated factors of HG. According to famous 50s rule of thumb, 50% of the population has at least 50% gray hair at 50 years old, but Panhard et al. reported that 6–23% of the population has at least 50% gray hair at 50 years old.^4^, ^18^ In a previous study conducted around the world, it was reported that incidence of HG in same age groups was very low in sub-Saharan African and African-Americans than other ethnic origins and severity of HG was the lowest in the African and Asian groups and the highest in the European group.^18^ The frequency and severity of HG increase with age, regardless of ethnic origin and geographical factors. In our study, the prevalence of HG was 67.2% in the 4th decade, 85.2% in the 5th decade, 97.3% in the ≥51 years. Similar to European groups, our results were higher than the results of the study in Koreans.^19^

HG usually starts at around 40 years but it may start at any age.^1^, ^19^ In a previous study including Korean participants, the mean age of onset of HG was 41.6 ± 13.1 years and was similar in females and males were reported.^19^ In our study, the mean age of onset of HG was lower (mean 32.9 ± 9.8 years) and it was lower in females than males. These results indicate that the prevalence and age of onset of HG may vary according to gender, ethnic and geographical origin. Similar to literature, severity of HG was significantly higher in males and also prevalence of HG was higher in males but there was no statistically significant difference in our study.^18^

In our study, the most common involved area of HG at onset and at present were parietal region in females and temporal region in males. Occipital region was the least involved area in both genders. These results were similar to the results of the previous study by Jo et al. but there are some different results in literature too.^3^, ^18^, ^19^, ^20^

If HG starts before the age of 20 years in Caucasians and before 30 years in African American population, it is considered as early-onset HG.^4^ It was reported that HG was more progressive in the late-onset group than in the early-onset group and this did not mean that the early onset would be a rapid progress in a previous study.^19^ Similarly, severity of HG was higher in the late-onset group than in the early-onset group in our study. The most common involved area of HG at the onset and the present was parietal region in participants with early-onset HG; whereas it was temporal region in participants with late-onset HG in our study, unlike the results of previous study.^19^ Additionally, the most common involved area of HG at the onset and the present were temporal in participants with severe HG; whereas parietal in participants with mild HG in our study. According to these results, male gender, late-onset and temporal onset may be considered to be poor prognostic factors for HG; but this data should be supported by new studies.

Genetic, environmental factors, nutritional status and oxidative stress play roles in the etiopathogenesis of HG.^2^, ^3^, ^4^ Early-onset HG is more common in individuals with a family history of early-onset HG.^5^, ^6^, ^7^, ^8^ In our study, the risk of HG was 5 times higher in participants with a family history of early-onset HG. This result once again showed that the importance of genetic factors in etiopathogenesis of HG. Previous studies have reported that smoking, alcohol consumption and obesity may be associated with early-onset HG.^5^, ^6^, ^7^, ^8^, ^9^ There was no relation between smoking and HG; but HG was more frequent in obese participants in our study. There are conflicting results between alcohol consumption and HG in the literature.^7^, ^19^ However alcohol consumption was lower in participants with HG in our study. This result may be related to low alcohol consumption in the especially elderly Turkish population in living rural areas.

Psychological disorders such as anxiety and depression may play roles in the etiopathogenesis of HG by increasing oxidative stress.^21^, ^22^ It was reported that the perceived stress was much higher in participants with early-onset HG.^6^, ^7^, ^8^ In our study, anxiety was more frequent in HG group; but there was no difference in the frequency of depression and stress.

In addition educational status and monthly income were lower in participants with HG. Living conditions are more difficult in people have lower socioeconomic status. This may lead to HG by increase oxidative stress in people. HG was more frequent in participants with darker skin type in our study. Its cause may be easy detection of HG in participants with darker skin type. Other than this, HG was more frequent in married, widow and having hair loss people. Its reason can be explained by age.

Aging and chronic diseases may cause HG by increasing oxidative radicals.^23^ HG, particularly early-onset HG is associated with various systemic diseases such as coronary artery diseases, osteopenia and hypothyroidism.^11^, ^12^, ^13^, ^20^, ^24^, ^25^, ^26^ Erdoğan et al. suggested that the presence of early-onset HG may be useful in identifying individuals at risk for cardiovascular diseases.^24^ In addition, it was suggested that HG may be an indicator of susceptibility to atherosclerosis and other advanced age-related diseases.^27^ In our study, HG was more common in participants with chronic diseases and the most common concomitant systemic diseases were hypertension, thyroid diseases and cardiovascular system diseases, respectively.

There were limitations of our study. It was performed in only Turkish individuals so this result cannot be generalized. The recall biases particularly for the age of onset of HG, the most common involved area of HG at the onset and family history of early-onset HG were another limitations.

## Conclusion

In conclusion, the prevalence of HG was higher and the mean age of onset of HG was lower than literature in our study. The mean age of onset of HG was lower in females than males, however severity of HG was higher in males. The most common involved area of HG was parietal region in females and temporal region in males. Occipital region was the least involved area in both genders. Severity of HG was higher in the late-onset HG. The most common involved area of HG at the onset and at the present was temporal region in severe HG; whereas parietal region in mild HG. Consequently, male gender, late-onset and temporal onset may be considered to be poor prognostic factors for HG. There is need for further epidemiological studies in people with different ethnic origin to illuminate the clinical and epidemiological characteristics and associated factors of HG.

## Financial support

None declared.

## Authors’ contributions

Ersoy Acer: Statistic analysis; approval of the final version of the manuscript; conception and planning of the study; elaboration and writing of the manuscript; obtaining, analysis, and interpretation of the data; effective participation in research orientation; intellectual participation in the propaedeutic and/or therapeutic conduct of the studied cases; critical review of the literature; critical review of the manuscript.

Didem Arslantaş: Statistic analysis; approval of the final version of the manuscript; conception and planning of the study; elaboration and writing of the manuscript; obtaining, analysis, and interpretation of the data; effective participation in research orientation; intellectual participation in the propaedeutic and/or therapeutic conduct of the studied cases; critical review of the literature; critical review of the manuscript.

Gülsüm Öztürk Emiral: Statistic analysis; approval of the final version of the manuscript; conception and planning of the study; elaboration and writing of the manuscript; obtaining, analysis, and interpretation of the data; effective participation in research orientation; critical review of the manuscript.

Alaattin Ünsal: Approval of the final version of the manuscript; conception and planning of the study; obtaining, analysis, and interpretation of the data; critical review of the literature; critical review of the manuscript.

Burcu Işıktekin Atalay: Statistic analysis; approval of the final version of the manuscript; conception and planning of the study; elaboration and writing of the manuscript; obtaining, analysis, and interpretation of the data; effective participation in research orientation; critical review of the literature.

Saniye Göktaş: Approval of the final version of the manuscript; conception and planning of the study; obtaining, analysis, and interpretation of the data; effective participation in research orientation; critical review of the literature.

## Conflicts of interest

None declared.
